# Active microrheology determines scale-dependent material properties of *Chaetopterus* mucus

**DOI:** 10.1371/journal.pone.0176732

**Published:** 2017-05-31

**Authors:** W. J. Weigand, A. Messmore, J. Tu, A. Morales-Sanz, D. L. Blair, D. D. Deheyn, J. S. Urbach, R. M. Robertson-Anderson

**Affiliations:** 1 Department of Physics and Biophysics, University of San Diego, San Diego, California, United States of America; 2 Marine Biology Research Division, Scripps Institution of Oceanography, La Jolla, California, United States of America; 3 Department of Physics and Institute for Soft Matter Synthesis and Metrology, Georgetown University, Washington DC, United States of America; University of Michigan, UNITED STATES

## Abstract

We characterize the lengthscale-dependent rheological properties of mucus from the ubiquitous *Chaetopterus* marine worm. We use optically trapped probes (2–10 μm) to induce microscopic strains and measure the stress response as a function of oscillation amplitude. Our results show that viscoelastic properties are highly dependent on strain scale (*l*), indicating three distinct lengthscale-dependent regimes at *l*_*1*_ ≤4 μm, *l*_*2*_≈4–10 μm, and *l*_*3*_≥10 μm. While mucus response is similar to water for *l*_*1*_, suggesting that probes rarely contact the mucus mesh, the response for *l*_*2*_ is distinctly more viscous and independent of probe size, indicative of continuum mechanics. Only for *l*_*3*_ does the response match the macroscopic elasticity, likely due to additional stiffer constraints that strongly resist probe displacement. Our results suggest that, rather than a single lengthscale governing crossover from viscous to elastic, mucus responds as a hierarchical network with a loose biopolymer mesh coupled to a larger scaffold responsible for macroscopic gel-like mechanics.

## Introduction

Mucus is a ubiquitous viscoelastic gel present in all animals that protects against foreign pathogens and parasites [1–3], coats organs, and aids in digestion [4,5], while allowing oxygen diffusion and the passage of nutrients past the protective barrier that it can form [6]. Filter-feeder marine animals, such as the *Chaetopterus* marine worm, also use mucus to trap and filter food, deter prey and build housing tubes [7–9]. The myriad of essential mechanical roles that mucus plays are critically dependent on its rheological properties. In fact, the most debilitating human diseases that involve mucus, such as cystic fibrosis, are associated with changes in mucus rheological properties. Further, the multifunctional role of mucus, allowing for simultaneous mobility of nutrients at the microscale and coating and lubrication at the macroscale, suggests that the rheological properties vary over different lengthscales, allowing for both fluid-like and elastic responses at different scales. While fluid-like mechanics can allow for efficient particle transport; elastic, gel-like mechanics result in markedly hindered particle mobility and even trapping.

The multifunctional properties of mucus likely stem from the complex structure of the biopolymer mesh comprising the mucus. Mucus glycoproteins (mucins), lipids, ions, proteins, cells, cellular debris, and water are all components of mucus, with mucins playing the most significant role in the physical properties of the mucus [10–12]. Due to the important and complex role that both mechanics and structure play in mucus function, a number of researchers have investigated the rheological and structural properties of mucus from a range of animals and organs, with an underlying goal of elucidating the mechanisms affecting human mucus properties [13–16]. While the molecular structure of mucus in all animals and organs is somewhat similar, different studies have reported wide-ranging results [5,13,16–22].

Here we use active microrheology to characterize both the structure and scale-dependent rheological properties of mucus produced by the *Chaetopterus* marine worm. Previous macrorheology measurements on this mucus show that the viscous stress response is comparable to human mucus [23]. Further, the worms excrete ~10–100 μl of mucus upon modest mechanical stimulation, allowing for the collection of reliably clean mucus samples. By contrast, acquiring high quality, reproducible samples from "standard" sources (e.g. human, bovine, porcine, etc) is a significant challenge [24].

In our approach, we sinusoidally drive microspheres of various sizes (2–10 μm) and measure the resulting force acting on the probe. From these measurements we determine the storage modulus, *G′*, loss modulus, *G"*, and complex viscosity, *η* of the mucus. We find three rheologically distinct regimes in the worm mucus for *l*_*1*_ < 4 μm, *l*_*2*_ ~4–10 μm, and *l*_*3*_ >10 μm. For *l*_*1*_ the stress response is comparable to water, suggesting that the probes move largely within the pores of the mucus network. For *l*_*2*_, mucus exhibits mechanics indicative of a continuum material with loose entanglements and predominantly viscous response properties. The macroscopic elastic gel properties measured at the macroscale only arise for *l*_*3*_, where stiffer structures force the probes out of the optical trap. Our results shed important new light onto how mucus is capable of simultaneously performing a range of functions that require different material properties.

## Background

Both macro- and micro- rheological methods have been used to investigate the material properties of mucus [24]. Both techniques are able to extract viscoelastic properties including the storage modulus, *G′*, loss modulus, *G"*, and viscosity, *η*. Macrorheology applies macroscopic strains to a material and measures the bulk stress response, while microrheology uses micron-sized probes embedded in the material to determine material properties. While microrheology measurements use Stokes-Einstein relations to relate probe motion to material properties, this method relies on the assumption that the material can be treated as a continuum. In this continuum limit, the measured material properties are independent of the size of the probe and are comparable to the bulk measured properties. However, biopolymer networks such as mucus have a range of important intrinsic lengthscales, such as polymer persistence lengths and network mesh sizes, that prevent this continuum limit from accurately describing the behavior at all lengthscales. In such networks the continuum limit is generally understood to be achieved for probes larger than the characteristic mesh size of the molecular network. Probes smaller than the mesh size travel largely within the pockets or pores of the molecular mesh and thus principally characterize the Newtonian fluid surrounding the mucus network. However, this naïve assumption has been called into question by a number of studies that have reported larger correlation lengthscales in biopolymer networks beyond the mesh size [24,25]. Measurements carried out with a range of probe sizes can not only measure rheological properties over a range of lengthscales, but can also quantify the intrinsic correlation lengths or entanglement spacings present in the material.

Macrorheology experiments have reported viscosities of human mucus to be 10^4^−10^6^× that of water and to exhibit shear thinning at high shear rates [26–30]. Macroscopic measurements of *G′* and *G"* also show that most mucus systems behave as elastic-like gels rather than viscous liquids [17,31–38]. These results are in contrast to several microrheology studies which show that mucus responds as a purely viscous Newtonian fluid [5,38–42]. Passive microrheology studies have focused on identifying the mucus mesh size by determining the probe size below which probe transport mimics that of probes traveling through water, reporting a wide range of results [5,41,43–47], stemming from different mucus sources, different probes, and varying methods for passivating the diffusing probes to prohibit binding to mucus. Passive microrheology studies on human cervicovaginal mucus reported an average pore size of ~340 nm with a wide range of 50–1800 nm [43], while other groups report a range of 200–370 nm [44]. Authors suggest that this wide range indicates that the mucins can bundle and create cables that are ~3× thicker than individual mucin fibers [43,45]. In contrast, two studies on human cervical mucus, using FRAP and fluorescence imaging, found that for ~10 nm particles the viscous drag that the probes experienced was similar to that found in water while 180 nm probes were slowed by a factor of ~100 as compared to the drag experienced in water [41,46]. FRAP has also been used to measure the diffusion of both linear and supercoiled DNAs in bovine cervical mucus and infer a mucus pore size of ~12.5 μm [47], in agreement with pore size estimates of 1–20 μm, measured using electron [48] and confocal microscopy [49]. Alternatively, electron microscopy measurements on human mucus measure pore sizes of 100–380 nm [46].

Active microrheology, which can probe larger lengthscales and exert higher forces than passive techniques, has been used far less than passive microrheology to probe mucus systems. Recent active measurements of zebra fish intestinal mucus reported purely viscous response with viscosity ~5× that of water for lengthscales up to ~10 μm [5]. Conversely, active studies on pulmonary mucus reported evidence of a stiff scaffold with a mesh size on the order of microns which authors infer from 1 μm optical trap oscillations in which ~80% of probes could not stay trapped during oscillation [45]. Corresponding electron microscopy measurements revealed large heterogeneities in pore size ranging from ~500 nm to ~10 μm [45].

## Materials and methods

The *Chaetopterus* marine worms (a common, non-endangered species also known as the “parchment tube worm”) used in this study were collected as previously described.[50] In short, bundles of tubes were collected at ~10–20 m depth by SCUBA diving in the La Jolla Canyon, off the Marine Protected Area of the Scripps pier (between 32.871844/-117.261911 and 32.866052/-117.267860), under collection permit #SC4564 (to Scripps Institution of Oceanography (SIO) collector; California Department of Fish and Wildlife). The bundles of tubes were then maintained in open water circulation in the Experimental Aquarium Facility of the Marine Biology Research Division, SIO. The worms remained inside their tubes during this entire process (collection and transfer to aquarium). After a few days to a couple of weeks, bundles of tubes (and their worm inhabitants) were transferred in buckets filled with cold seawater for the quick transit (<10 km) between SIO and the University of San Diego (USD). The bundles of tubes were then stored at USD in filtered artificial seawater at ~15°C, under aeration, filtration, and good water circulation, until the worms were collected and manipulated for analysis of their mucus. Similar storage facilities were maintained at Georgetown University for the presented macrorheology experiments. This study was conducted in accordance to the 1966 Animal Welfare Act, and followed standard animal maintenance and manipulations ethics code of the University of California.

**Fig 1:**
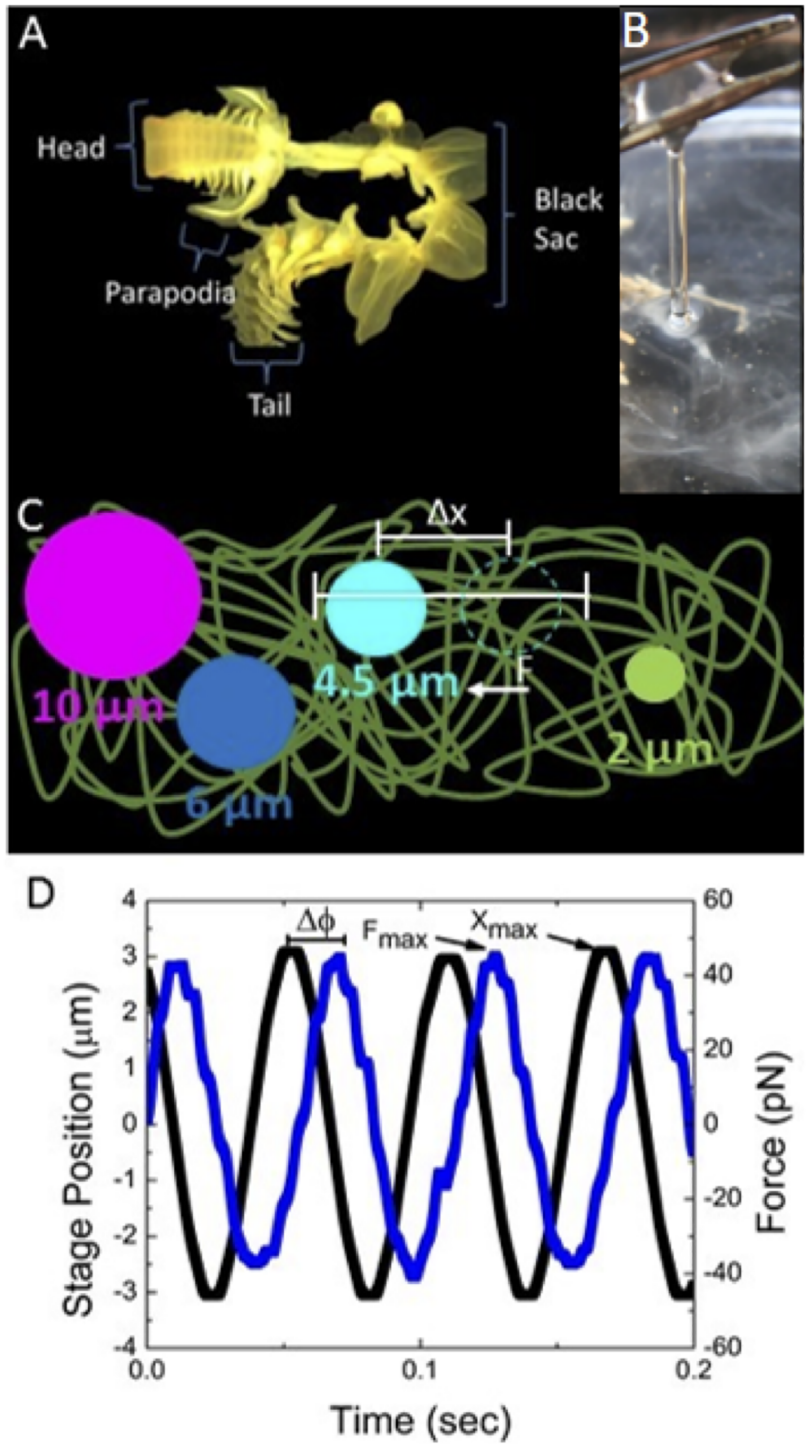
Schematic of active microrheology measurements of *Chaetopterus* marine worm mucus. (A) Typical *Chaetopterus* worm used for experiments, with body parts labeled. Mucus is extracted from the head and parapodia. (B) Image of viscoelastic mucus excreted by the worm. (C) Schematic of microspheres embedded in the mucus. Bead sizes are labeled and colored as they appear in the rest of the figures. Here, the 4.5 μm bead is trapped and sinusoidally oscillated at an amplitude Δ*x* while the force *F* on the bead is measured. (D) Sample force *F* (blue) and stage position *x* (black) curves are shown for a 6 μm bead. *F*_*max*_ is the amplitude of the force exerted by the mucus on the bead, *x*_*max*_ is the oscillation amplitude, and *Δϕ* is the phase difference between the force and stage curves.

The worm mucus extraction followed a similar method to that used previously [50]. We removed a worm from its housing tube, placed it on a petri dish with a small amount of seawater, and using a sharp blade, separated the posterior end (tail and black sacs) from the rest of the body. (The tail contains gonads and mucus from the tail usually contains gametes, which we wanted to avoid having in our mucus samples.) The anterior part of the organisms, which consists of the head and parapodia (Fig 1), was the focus of this study since it is known to produce a large amount of mucus. Mucus from the head and parapodia was extracted by applying light pressure to the body parts using a syringe and collecting the excreted mucus, which was stored at 4°C for up to 1 hour. Typical mucus samples contained 0.65 mg/mL carbohydrates and 1,045 mg/mL proteins, measured using GC Mass Spectrometry and BioRad DC Protein Assay, respectively. The dry weight was ~0.8% of wet weight. Because the mucus is excreted rather than scraped from epithelial layers, we expect there to be a negligible amount of shed epithelial cells in mucus, and no previous studies characterizing the biochemistry of the mucus have found evidence of cells in the mucus [7,51–54]. There is also no evidence from these studies or our own of DNA or bacteria present [7,51–54].

Carboxylate microspheres (beads; Polysciences) of 2, 4.5, 6, and 10 μm (each with <5% polydispersity) were coated with Alexa-Fluor 488 bovine serum albumin (Invitrogen) to prevent nonspecific binding to mucus and visualize the beads. For all experiments, we mixed 3 μL of 0.5% (v/v) beads with 10 μL of mucus and 0.015% Tween-20 (Fisher Scientific), which prevents beads from binding to the mucus and microscope slide. We then used a Pasteur pipet to transfer the mucus mixture into a microchannel comprised of a microscope slide and coverslip separated by two layers of double-sided tape, and sealed the ends with epoxy. Mixing was achieved by vortexing the sample for ~2 s. To ensure vortexing did not disrupt or modify the mucus structure, we carried out measurements on samples vortexed for 0.5–30 s, and compared to those mixed gently by pipetting the sample up and down with a wide-bore pipet tip. We found no appreciable difference between mixing methods, suggesting that the mucus structure we probed closely resembled that of the unperturbed state.

During measurements, individual microspheres were trapped using a custom built optical trap formed by a 1064 nm Nd:YAG fiber laser (Manlight) focused by a 60× 1.4 NA objective (Olympus). The sample was precisely oscillated sinusoidally relative to the trapped bead by using a piezoelectric nanopositioning stage (Mad City Laboratories). A position-sensing detector (PSD, Pacific Silicon Sensors) was used to measure the laser deflection, which is proportional to the force *F* on the microspheres. The force-detection was calibrated for each bead size using the Stokes drag formula for a microsphere oscillating in water [55], as well as the equipartition method in water and in mucus [56,57]. Oscillatory measurements in water were used to ensure that the trapping forces were consistent over our entire frequency and amplitude range, resulting in forces consistent with the Stokes drag formula and viscosities that were largely independent of amplitude, frequency and bead size (S1 Fig). However, because slight variations in the index of refraction of mucus compared to water could affect the magnitude of calibration values we also used the equipartition method to calibrate the trap directly in the mucus. The Brownian data recorded for this calibration method also demonstrate the symmetry and Gaussian profile of the trap (S2 Fig).

Following measurements, we fit both the measured force *F* and stage position *x* data to sine curves using the least squares method as described previously [58]. The fits were then used to calculate the storage modulus *G′ = [|F*_*max*_*|cos(Δϕ)]/[|x*_*max*_*|6πR]*, loss modulus, *G" = [|F*_*max*_*|sin(Δϕ)]/[|x*_*max*_*|6πR]*, and complex viscosity, *η = (G′*^*2*^ *+ G"*^*2*^*)*^*1/2*^*/ω* where *|F*_*max*_*|*, *|x*_*max*_*|*, *Δϕ* and *R*, are the amplitude of measured force, amplitude of the stage position, phase difference between *F* and *x*, and probe radius respectively [58]. Five trials, each averaging 10 full oscillations, were completed for each frequency, amplitude, and probe size. For each trial, a different bead in a different region of the sample was used. All viscoelastic moduli data shown are averages over mucus from three different worms with error bars representing the corresponding standard error. We carried out measurements for oscillation amplitudes of 1–8 μm and frequencies of 1–15 Hz.

For macrorheology measurements, mucus samples were also prepared from the head-parapodia body part of freshly-collected worms. Because a larger amount of mucus was needed for these measurements, the head-parapodia body part was placed into a plastic syringe, and gently compressed with the syringe plunger (to avoid excessive crushing of internal tissues). This mechanical forcing triggered mucus secretion that was collected drop by drop out of the syringe, while the body part was retained in the barrel of the syringe. The mucus collected from the syringe was mixed with water to produce a 50% v/v solution. We used a rotational rheometer (Anton Paar) equipped with a 25 mm diameter cone plate tool to exert oscillatory strain on the mucus solution and measure its elastic and loss moduli. The strain on the mucus started at 0.01% and slowly increased to 5%. The oscillatory frequency was kept constant at 1 Hz. Macrorheology data shown is an average of two individual worms and corresponding standard error.

We chose to passivate probe surfaces with BSA and Tween because we had previously found this method to prevent binding to biopolymers such as DNA and actin [59,60]. However, to test passivation of BSA-coated beads we also carried out particle-tracking measurements with 2 μm and 6 μm beads coated with BSA, 2 kDa PEG-diamine (which has been shown to result in mucoinert probes [39,61]) and streptavidin (a “sticky” protein with enhanced nonspecific interactions). We tracked ~30–40 probes diffusing in different regions of three different mucus samples, for a total of ~100 measurements. For each probe, we captured data at 10 fps for ~20 s using an Orca Flash 2.8 CMOS camera (Hamamatsu). We measured the mean-squared displacements in the *x* and *y* directions (*<Δx*^*2*^*>*,*<Δy*^*2*^*>*) and determined the resulting diffusion coefficients (*D*) via *<Δx*^*2*^*> = <Δy*^*2*^*> = 2Dt*. For each of the three mucus samples, all 4 probe types/sizes were embedded in the same sample for direct comparison of the effects of coating on diffusion. As shown in S3 Fig and described in Results, the transport properties of PEG-coated and BSA-coated probes in mucus were quite similar, suggesting similar passivation efficacy, whereas the diffusion coefficients of streptavidin-coated probes were significantly reduced.

## Results and discussion

**Fig 2:**
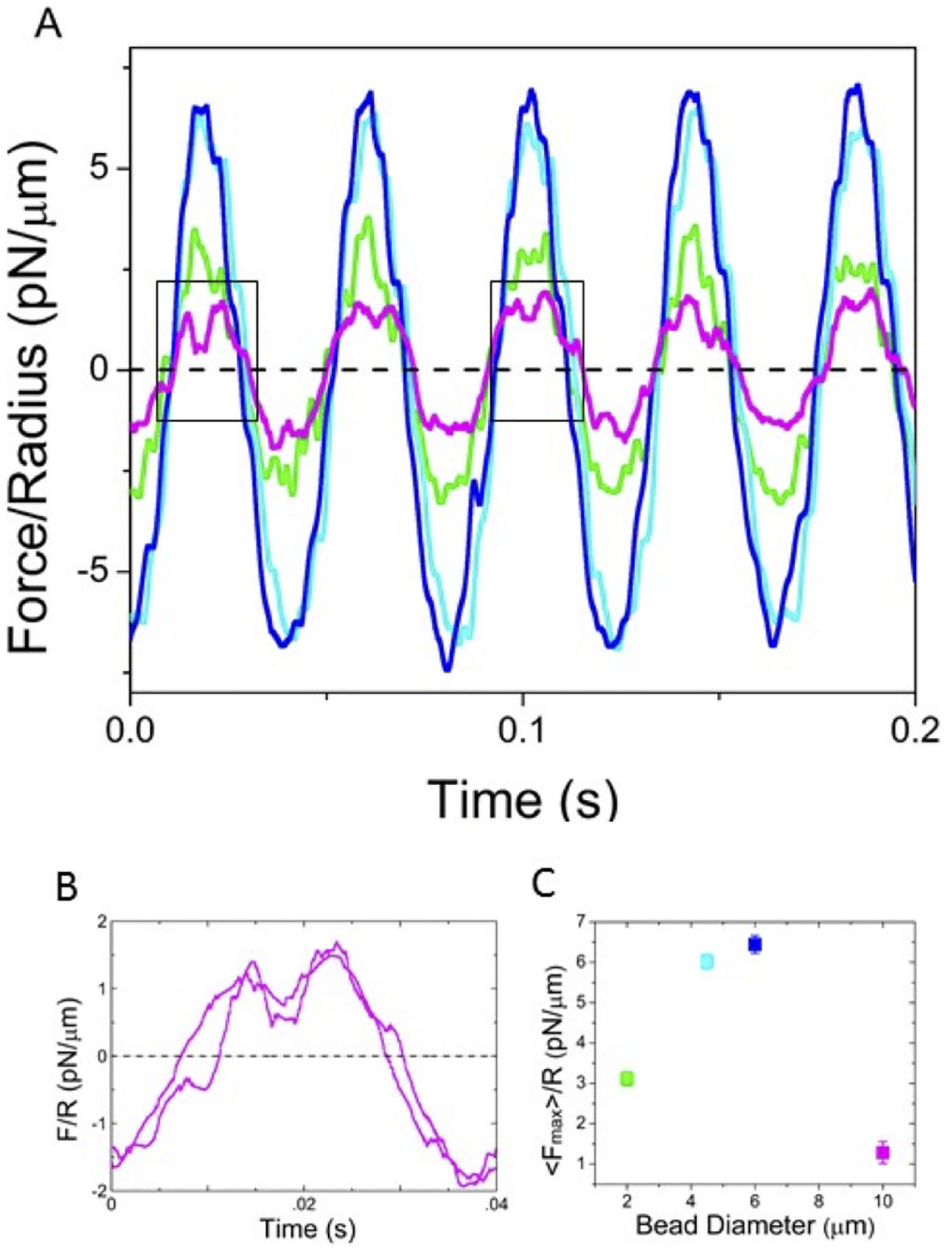
Force response reveals lengthscale-dependent material properties of mucus mesh. (A) Sample force curves normalized by corresponding bead radii (*F/R*) show scale-dependent material response. Force curves shown are for an oscillation amplitude of 3.6 μm and frequency of 10 Hz for probe sizes of 2 (green), 4.5 (cyan), 6 (blue), and 10 μm (magenta). *F/R* for 4.5 and 6 μm beads are nearly identical indicating the mucus is responding as a continuum fluid at these lengthscales (*l*_*2*_). The ~2**×** reduction in *F/R* for 2 μm probes indicates a transition to a water-like regime (*l*_*1*_). The ~3**×** reduced *F/R* for the 10 μm probe indicates that the bead is being forced out of the trap and does not track with the trap position during the complete oscillation. This forcing, unique to 10 μm probes, indicates the presence of stiffer structures at this lengthscale (*l*_*3*_). (B) The force peaks from the boxed regions in (A) show that *F/R* for 10 μm probes drop nearly to zero at the maximum position, as the bead is forced out of the trap. (C) The average normalized force amplitude, *<F*_*max*_*>/R*, as a function of bead diameter clearly shows the described distinction between the three different lengthscales (*l*_*1*_ (green), *l*_*2*_ (cyan, blue), *l*_*3*_ (magenta)).

As shown in Fig 2, oscillatory force measurements show a complex dependence on probe size. As described in Background, if the mucus responds as a continuum, the measured force *F* should be proportional to the probe size *R*, resulting in probe-independent material properties (i.e. *G′*, *G"*, *η* ~ *F/R*). For the 4.5 and 6 μm beads, the force response normalized by the probe size is indeed independent of probe size indicating that at these lengthscales the biopolymer mesh comprising mucus can be treated as a continuum material. In contrast, the 2 μm beads measure a ~2× lower normalized force indicating that they interact less with the mesh network. These discrete effects suggest that the transition to the continuum regime is ~4 μm. This lengthscale is substantially (~5×) larger than previous estimates measured via passive microrheology and microscopy in human and bovine mucus systems [41,43–46]. Thus, this finding suggests that the mesh is very loosely entangled so it only contributes appreciably to the force response due to active perturbations for lengthscales ~5× the mesh size. These results are in agreement with our previous results for entangled DNA in which we determined that the transition to continuum occurred at ~5× the entanglement spacing of the network [25]. However, mucus mesh size estimates as high as 20 μm have been made based on microscopy methods and theoretical predictions [23,47,50,62,63], suggesting that there are perhaps multiple lengthscales that control mechanics of mucus systems.

**Fig 3:**
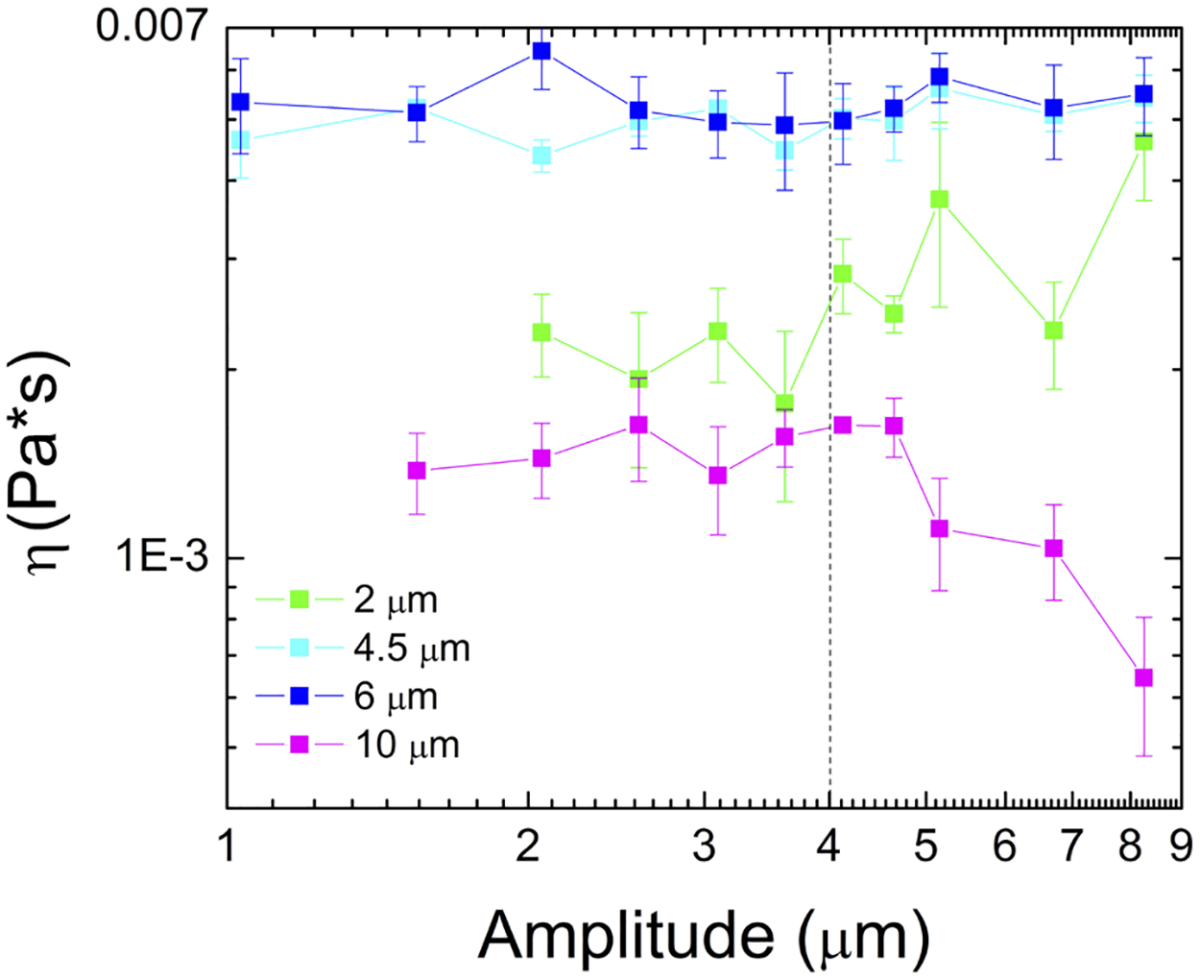
Viscosity vs oscillation amplitude reveals a critical lengthscale of ~4 μm controlling mechanics. Viscosity, *η* (Pa-s), as a function of oscillation amplitude for 2 (green), 4.5 (cyan), 6 (blue) and 10 μm (magenta) probes. Note that the viscosities for 4.5 and 6 μm probes are nearly identical. Smaller *η* values for 2 μm probes indicate a water-like regime (*l*_*1*_) which only transitions to the continuum regime for amplitudes >4 μm (*l*_*2*_, dashed line). The reduced *η* for 10 μm probes is an artifact of the probe being forced out of the trap, likely by larger stiffer structures in the mucus (*l*_*3*_).

**Fig 4:**
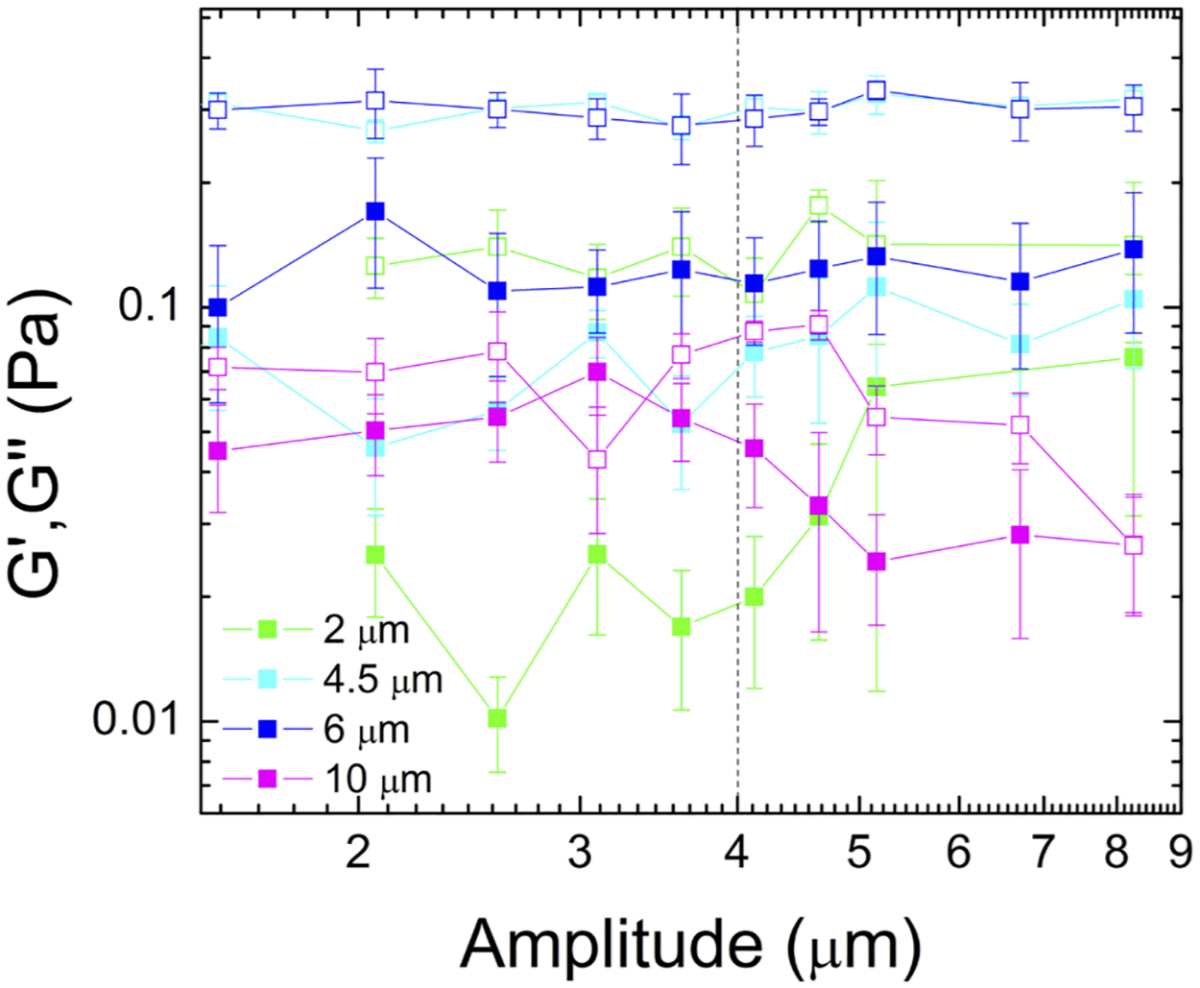
Frequency-dependent viscoelastic moduli indicate that the mesoscale polymer mesh is highly viscous while elasticity is a largescale phenomenon. Elastic modulus, *G'*, (closed squares) and viscous modulus, *G''*, (open squares) as a function of amplitude for four different probes sizes. Note the similar values for the 4.5 and 6 μm beads and the increase in *G′* for the 2 μm probe at *x*_*max*_ > 4 μm. The reduced gap between *G′* and *G"* for 10 μm probes indicates a more elastic response.

Indeed, contrary to the typical single fluid model, in which the response is independent of bead size above one critical lengthscale that defines the continuum regime [64], we find that for 10 μm beads the normalized force drops by a factor of ~3 when compared to the 4.5 and 6 μm beads (Fig 2). Upon closer examination we see that the force oscillation curves have relatively flat or sunken peaks rather than the expected sinusoidal response seen with the other probe sizes (Fig 2b). These dips in the force peaks demonstrate that the bead is forced out of the trap during oscillation and is picked up again as the stage returns to equilibrium. This forcing, which causes the bead to be “free” for ~40% of the oscillation time (Fig 2b), is likely due to the probe encountering an elastic network or structure that is strong enough to force it out of the trap [45]. Resulting viscoelastic moduli calculated from the truncated force curves (Figs 3 and 4) will consequently have smaller amplitudes, as these moduli are proportional to *|F*_*max*_|. Previous studies in multiple mucus systems have also shown evidence of a similar stiff elastic scaffold at larger lengthscales, likely due to mucin bundling and crosslinking [43,45,65].

These results suggest that the mucus is a “multiscale mesh” with multiple distinct lengthscales. To test this hypothesis and characterize the mechanical properties at each of these lengthscales, *l*_*1*_ ≤ 4 μm, *l*_*2*_ ≈ 4–10 μm, and *l*_*3*_ ≥ 10 μm, we turn to the amplitude, frequency and probe size dependence of the resulting viscoelastic properties derived from the measured force response.

The complex viscosity as a function of amplitude and probe size supports this hypothesis (Fig 3). For 4.5 and 6 μm probes the viscosities are nearly identical, ~5× higher than that of water, for the entire amplitude range. For 2 μm probes the viscosity is relatively constant for small amplitudes and is only ~2× the viscosity of water. At an oscillation amplitude of ~4 μm there is a marked increase in the viscosity, approaching the corresponding values of the 4.5 and 6 μm probes, indicating a transition from a water-like regime to the continuum regime. Specifically, the amplitude-averaged viscosities for amplitudes <4 μm is 2.0 +/- 0.2 mPa-s while the average for >4 μm is 3.3 +/- 0.9 mPa-s, ~55% higher than the smaller amplitude data. The increased variance in data at the higher amplitudes is also consistent with this picture as the heterogeneous nature of the mesh would lead to a wider spread in data than for a probe traveling through water.

Our passive particle-tracking measurements for 2 μm and 6 μm probes support these findings (S3 Fig). We find that the diffusion coefficient in mucus (*D*_*m*_) for both probes is reduced from that in water (*D*_*w*_), but for 2 μm probes *D*_*m*_ ≈ *(0*.*70 ± 0*.*11)D*_*w*_ while for 6 μm beads *D*_*m*_ ≈ *(0*.*24 ± 0*.*06)D*_*w*_. These results are consistent with the corresponding increased viscosities measured with the 2 μm and 6 μm probes (~2*η*_*w*_ and ~5*η*_*w*_ respectively), and show that while the transport of the smaller probes is only modestly influenced by the surrounding mucus mesh, the mucus imposes a significant barrier to diffusion of larger probes. Further, results are similar for both PEG-coated microspheres, which have been shown to be mucoinert [39,61], and BSA-coated microspheres, demonstrating that BSA acts an effective blocker to binding interactions with the mucus.

If the mucus had a single correlation length that controlled the onset of continuum mechanics, then the viscosity for the 10 μm beads would be the same as that for the 4.5 and 6 μm beads. In contrast, we find that the viscosity measured with the 10 μm beads is even lower than the 2 μm beads. This artificially low viscosity is an artifact of the resistive force of the mucus network forcing the bead out of the trap (resulting in low measured forces). Similar to the 2 μm beads, the apparent viscosity is constant up to ~4 μm after which it drops considerably because the probe cannot follow the trap for the full oscillation. Because *η~F*_*max*_*/x*_*max*_, if the measured force amplitude *F*_*max*_ remains constant due to the bead being forced out of the trap at a specific strain distance by a stiff structure in the mucus, then as the amplitude *x*_*max*_ increases, *η* will decrease accordingly.

To further test this hypothesis we carried out measurements in a 50% dilution of mucus (in water). We find that upon dilution the average measured viscosity for the 2 μm beads was reduced to 79% of the value for 100% mucus, while for 10 μm beads the viscosity at 50% dilution was *increased* by 15%. The measured viscosities for the 4.5 and 6 μm beads were both reduced to ~95% of the 100% mucus value. This interesting result corroborates with the fact that the measured viscosity for the 2 μm beads is due to the polymer mesh, which is reduced at lower mucin concentrations, while the increased viscosity of the 10 μm beads is likely a result of elastic structures (e.g., bundled mucins) occurring less frequently and/or having less rigidity (fewer mucins bundled together) at lower mucin concentrations. We note that these results suggest only a modest increase in the threshold lengthscale *l*_*2*_ upon 50% dilution, as the viscosities for the 4.5 μm and 6 μm beads are not reduced significantly. Our results instead indicate that the principle effect of reducing the mucus concentration is to reduce mucin bundling, leading to an increase in or elimination of *l*_*3*_.

Within our hypothesized framework, we should also expect the mucus to respond more elastically at larger scales, which we can determine by comparing the elastic modulus, *G′*, to the viscous modulus, *G"* for each probe size (Fig 4). As expected, moduli for the 4.5 and 6 μm probes are nearly identical. Further, *G" ≈ 3G′* for these probe sizes over the entire amplitude range showing that the mucus response is largely viscous. Conversely, for 10 μm beads, *G′* and *G"* are comparable to each other, indicating that elasticity is playing more of a role in the response at these larger scales. Finally, for 2 μm beads *G"* is nearly an order of magnitude larger than *G′*, and only for amplitudes >4 μm do we see *G′* increase with increasing amplitude, approaching the values measured for 4.5 and 6 μm beads. This increase suggests that the viscous mesoscale mesh (*l*_2_) is coupled to the macroscale elastic-like scaffold (*l*_*3*_). In other words, because the 2 μm probe is still smaller than the continuum lengthscale (*l*_*2*_), the loosely entangled polymers could easily sweep past the probe as it moved through this mesoscale mesh unless they were coupled to the elastic scaffold. This coupling would provide elastic resistance that prevented the polymers from completely moving out of the way of the moving probe and provide an elastic restoring force. This coupling is likely achieved via steric entanglements or chemical crosslinking as suggested to occur in other mucus systems [43,45,47].

A similar “two-fluid” model has been used to describe the structure of human cervicovaginal mucus as measured using electron microscopy [45]. However, in these studies the material properties at different lengthscales were not characterized and coupling between the lengthscale-dependent structures was not explored. It is possible that the increased elasticity is a result of the 10 μm beads inducing bundling of the mucins or other structural changes to the mucus due to the substantial volume exclusion from the probe. However, the volume fraction of the probes in the mucus is <0.1% so the overall volume reduction or increase in effective concentration of mucins is essentially negligible and unlikely to contribute to the response.

**Fig 5:**
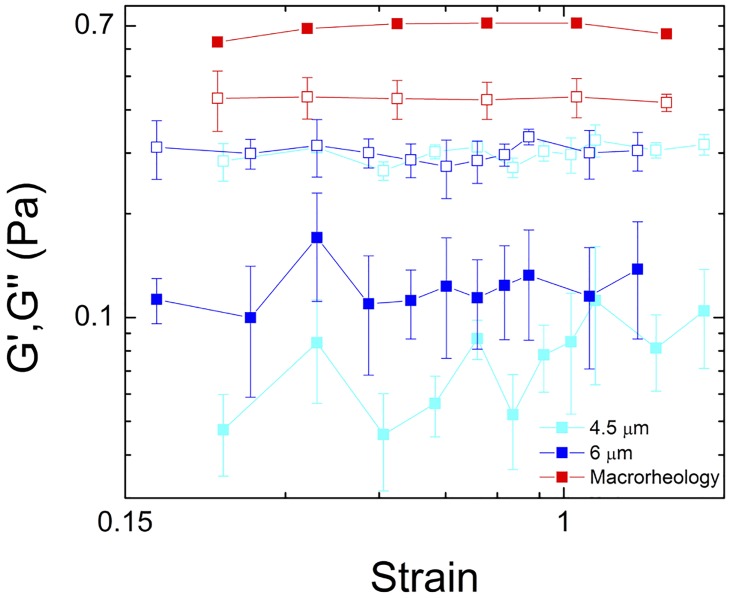
Elasticity dominates the macroscale response, while viscosity is comparable on multiple lengthscales. Elastic (closed) and viscous (open) modulus measured via macrorheology (red) and microrheology using 4.5 (cyan) and 6 μm (blue) microspheres. Macrorheology measurements exhibit *G′>G"* (in contrast to microrheology data) indicating that elasticity is a macroscale phenomenon. *G"* for both macrorheology and microrheology data are similar, indicating that the dissipative mechanics arise from the loosely entangled polymers in the mucus.

While our microrheology measurements demonstrate evidence of a stiff elastic network, the trap is not strong enough to hold the 10 μm probe and measure the full force response. So to characterize the perceived elastic mesh at large lengthscales, we compare our results with macrorheology measurements. We can quantitatively compare microrheology measurements to macrorheology data by converting the microrheology amplitude values to strain (*x*_*max*_*/2R*) (Fig 5) [59]. For macrorheology measurements, *G′* is larger than *G"* for all measured strains, indicating that elasticity is a property revealed on the macroscale. Elastic gel-like networks are generally comprised of stiffer structures including bundled filaments that are highly entangled or crosslinked; and previous studies on mucins and human cervicovaginal mucus suggest that mucin polymers can self-assemble and cross-link into stiff bundled fibers [43,45]. Because each bundle is comprised of many mucins, the resulting mesh size for such a network of stiff bundles would be substantially larger than the mesh size of an entangled mucin network of the same concentration [43,45]. Thus, this macroscale elasticity likely arises from this larger mesh of bundled polymers that is probed by the 10 μm beads. Conversely, the macroscopic *G"* is nearly the same as the microscale value for the 4.5 and 6 μm probes, indicating that the viscous response is the same at all scales and comes from the loosely entangled polymers that permeate the mucus and dominate the mesoscale mechanics (*l*_*2*_).

**Fig 6:**
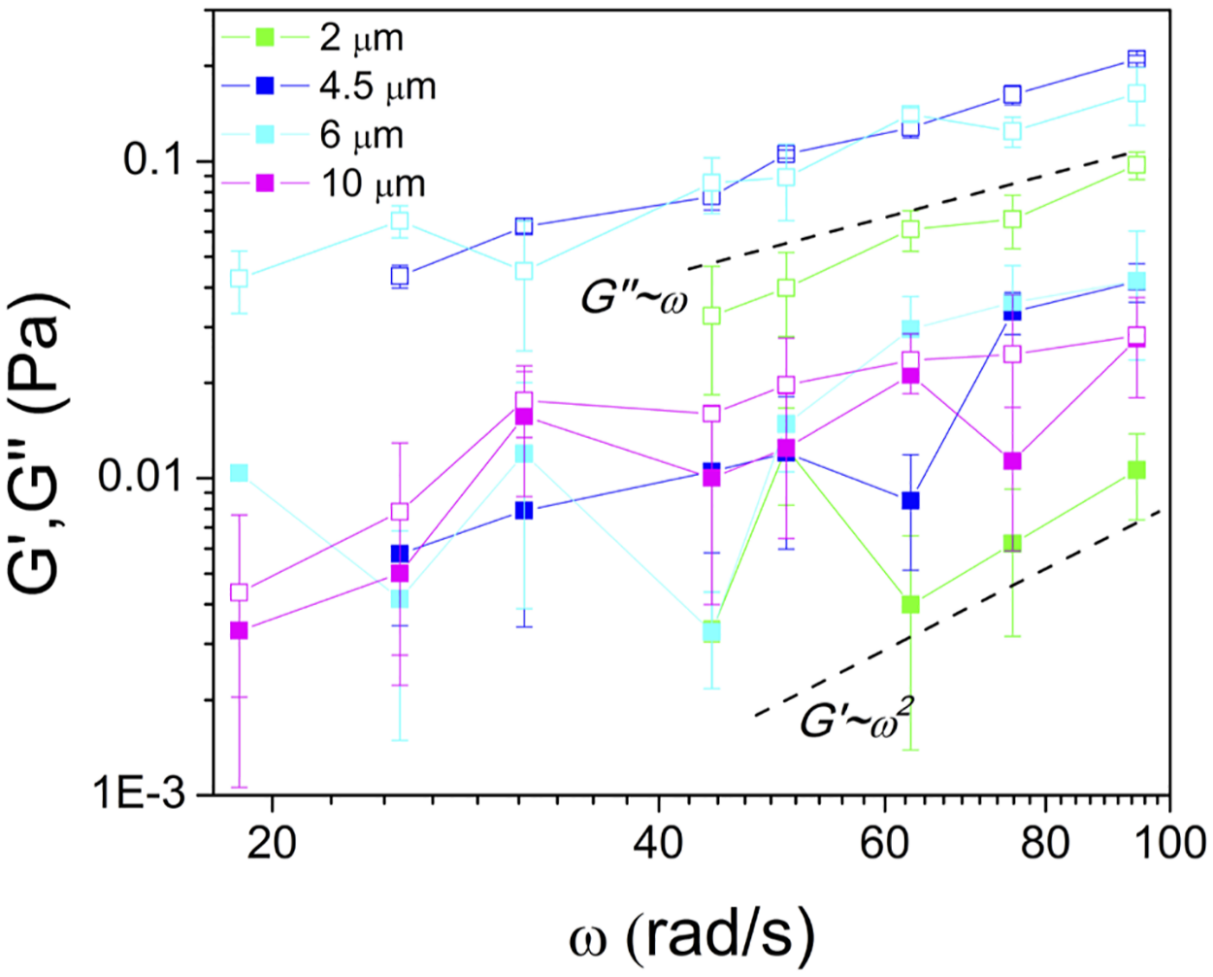
Lengthscale-dependent viscoelastic effects of mucus. *G'(ω)* and *G"(ω)* for 2 (green), 4.5 (cyan), 6 (blue), and 10 μm (magenta) probe sizes. The scaling of *G′* and *G"* for 2 μm beads indicates that the mucus is responding principally as a Newtonian fluid with minimal elasticity, indicated by the terminal regime scaling laws represented by dashed lines. For the larger probes, the scaling of both *G′* and *G"* deviate from terminal regime scaling indicating the onset of viscoelastic effects. Consistent with our proposed model, the values for 4.5 and 6 μm beads coincide (*l*_*2*_), and for 10 μm beads *G′* approaches *G"* and both *G′* and *G"* approach frequency-independent plateaus.

To further characterize the lengthscale-dependent material properties of the mucus we measure the frequency dependence of the viscoelastic moduli (Fig 6). For a network that exhibits little elasticity and flows largely like a Newtonian fluid (the terminal regime for entangled polymers), we expect *G′*~*ω*^*2*^ and *G"*~*ω*. As elastic effects become more important, i.e. as the frequency of perturbations approach and surpass the relaxation or disentanglement rate of network, *G′* approaches and surpasses *G"*, and both *G′* and *G"* initially approach frequency-independent plateaus. As shown in Fig 6, for 2 μm probes *G′*~*ω*^*2*^ and *G"*~*ω* while for 4.5 and 6 μm probes, the scaling of *G′* and *G"* is slightly reduced from terminal regime scaling and the magnitudes of *G′* and *G"* are once again probe size independent. For 10 μm beads, *G′* and *G"* both approach frequency-independent plateaus with a further reduced gap between *G′* and *G"*.

**Fig 7:**
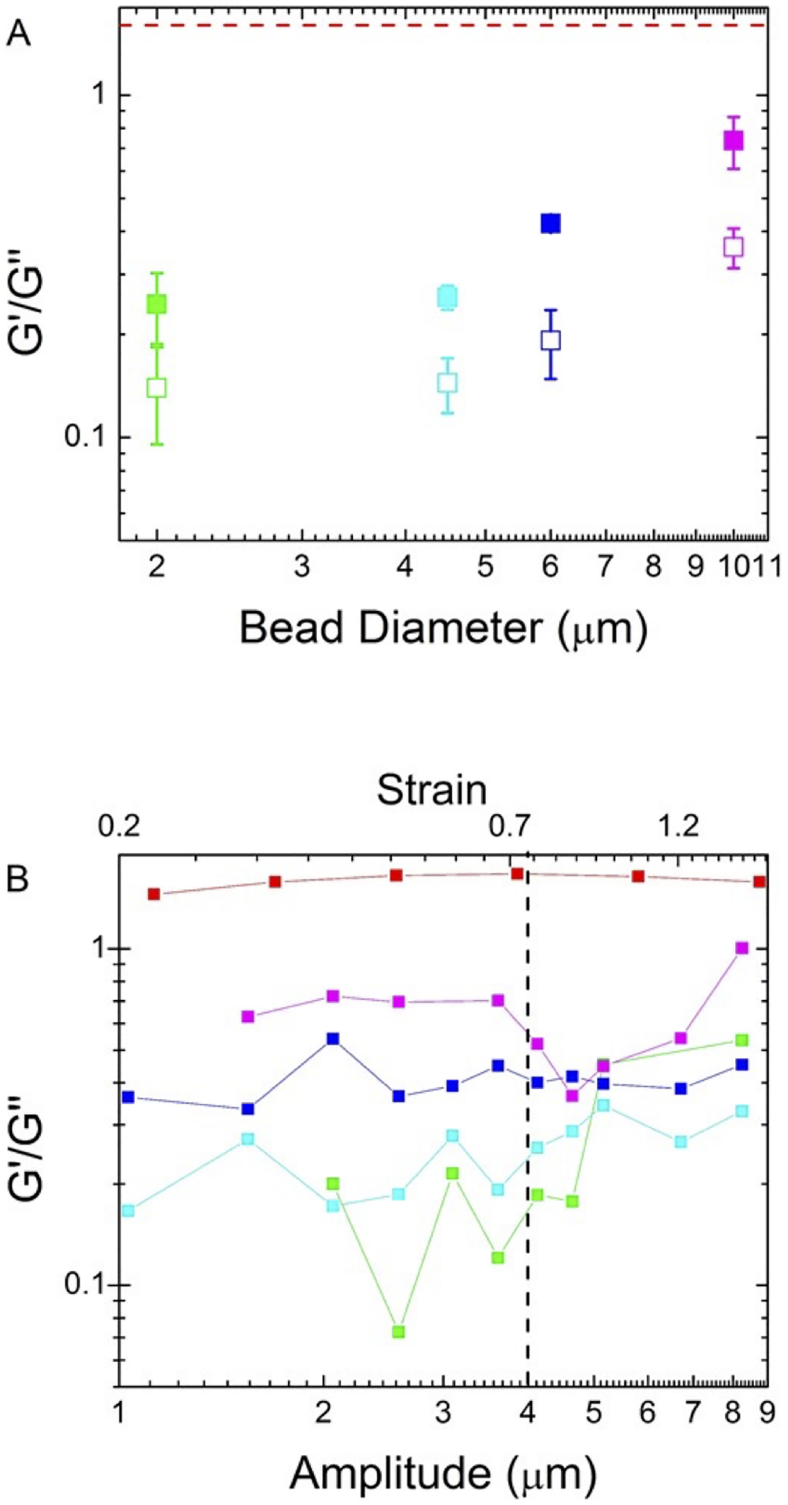
Relative elasticity <*G′/G"*> of mucus increases with increasing lengthscales. (A) Average relative elasticity <*G′/G"*> for 2 (green), 4.5 (cyan), 6 (blue), 10 μm (magenta) beads and macrorheology data (red). Closed squares are < *G′/G"*> values averaged over all amplitudes while open squares are averaged over all frequencies. The relative elasticity increases as probe size increases, approaching <*G′/G"*> for macrorheology data. (B) <*G′/G"*> as a function of amplitude shows that elastic effects begin to play a role in mechanics beyond 4 μm.

We quantify the relative elasticity of the mucus as a function of probe size by comparing the average ratio of the elastic modulus to viscous modulus <*G′/G"*> for all four bead sizes as well as for macrorheology. This quantity can also be understood as the inverse of the loss tangent, tan *δ* = sin*Δϕ/*cos*Δϕ*, which quantifies the relative dissipation in the system. Fig 7 shows that the relative elasticity increases with increasing bead size, and for 10 μm beads this ratio is comparable to the ratio of the macroscopic moduli, indicating that the stiff mesh responsible for pulling the 10 μm bead out of the trap is also what leads to the macroscale elastic response. *G′/G"* values for all probe sizes converge as oscillation amplitude increases, in line with our interpretation of a threshold lengthscale necessary for the mucus mesh to appreciably contribute to measured mechanics.

The results reported here are consistent with similar previous experiments with porcine gastric mucus in which passive microrheology with 1 μm beads measured *G′* and *G″* values nearly >50× smaller than macrorheology measurements, with *G′/G″* also substantially reduced [38]. We note that these results were only for low pH conditions in which the gastric mucus forms a gel. At higher pH when the mucus is principally a viscous solution, macrorheology and microrheology reported similar behavior. The authors explain this lengthscale-dependent rheological response in terms of a swollen heterogeneous gel of bundled mucins. Corresponding AFM images show that the swollen gel consists of extended mucins interspersed with nodes of bundled mucin clusters, appearing much like a pearl necklace [66]. While these results were specific to low pH conditions, such a structure would likely lead to rheological results similar to ours. Specifically, the perceived double-network could arise from a network of nodes of bundled mucins (*l*_*3*_) linking together entangled extended mucins (*l*_*2*_).

The lengthscale dependence of the mucus mechanics described here is distinct from the heterogeneous nature of mucus and many other biopolymer networks. Heterogeneities in network structures, leading to large variances in measurements taken in different regions of the material, have clearly been shown in a wide range of biopolymer networks [67–70]. The results we have presented instead focus on extensive lengthscale-dependent measurements with varying probe sizes, oscillation amplitudes and frequencies, using three different mucus samples (and 5 different measurements within each sample). However, prior to these measurements, to characterize the sample-to-sample and spatial heterogeneities of the mucus, we carried out similar oscillatory measurements in mucus samples from over 25 worms and in up to 20 different regions in the same mucus sample. These measurements showed a ~20% range in |*F*_*max*_| values for data collected in different mucus samples as well as in different regions of the same sample. However, the data trends with respect to oscillation amplitude and frequency were similar across all trials. This 20% heterogeneity is well above the ~5% polydispersity in nominal probe sizes and the ~2.6% instrument error in force detection (see S1 Fig), and is thus characteristic of the heterogeneity in the mucus itself. This 20% heterogeneity must be kept in mind when interpreting our results, as they represent average mechanical properties of the mucus but do not necessarily exactly match the mechanics of any single mucus sample.

## Conclusion

**Fig 8:**
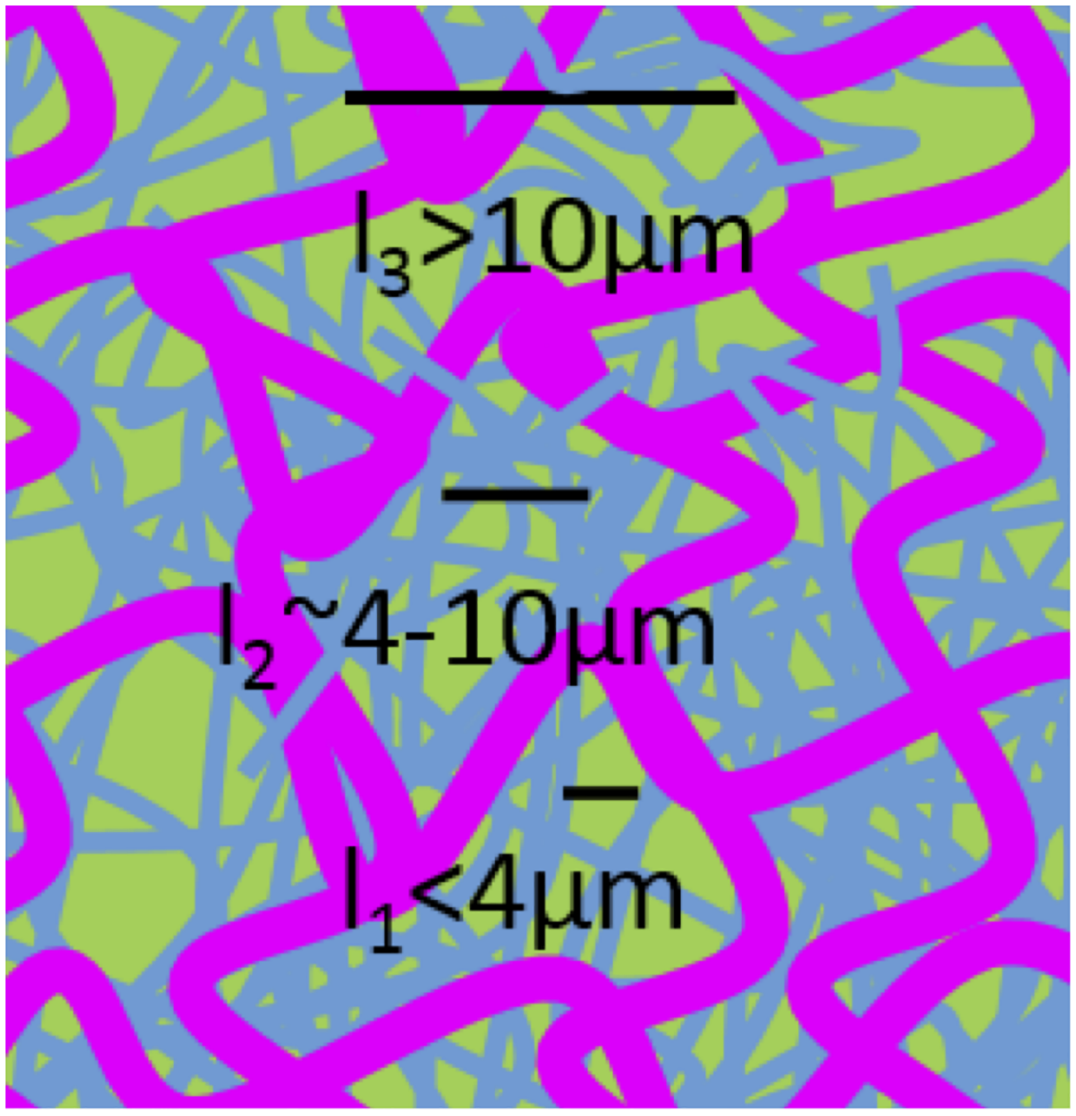
Lengthscale-dependent microrheology measurements suggest that mucus can be modeled as a coupled two-fluid system. Cartoon of the proposed structure of the marine worm mucus. *l*_1_ is the water-like regime, where the mesh plays little role in the response and particles can freely pass through the mucus. *l*_2_ is the mesoscale continuum regime comprised of loosely entangled polymers. *l*_*3*_ is the regime where the stiff scaffold produces the elastic-like macroscopic mucus properties. Coupling of the two meshes at *l*_*2*_ and *l*_*3*_ may be achieved via steric entanglements or chemical crosslinking.

We have used active microrheology to characterize the lengthscale-dependent structural properties governing the mechanics of *Chaetopterus* mucus. Depending on the lengthscale probed, the mucus can respond: similarly to water, with minimal contribution from the mucus mesh (*l*_*1*_); as a continuum with principally viscous response features, characteristic of a loosely entangled polymer mesh (*l*_*2*_); or elastically, mimicking the macroscopic mucus properties (*l*_*3*_). These collective results suggest that the mucus can be modeled as a coupled two-fluid system comprised of (i) a loosely entangled polymer mesh coupled to (ii) a larger more elastic scaffold (Fig 8). While mesh (i) is responsible for the enhanced viscosity of the mucus at lengthscales above *l*_*1*_, mesh (ii) provides the macroscale elasticity of the mucus at *l*_*3*_.

This model can explain how mucus is able to simultaneously perform a multitude of functions across varying lengthscales. For example, *l*_*1*_ is necessary for the passage of nutrients while *l*_*2*_ is needed for trapping larger pathogens and parasites. Finally, the macroscopic gel-like properties necessary for coating organs is provided by *l*_*3*_.

## Supporting information

S1 Fig Active microrheology measurements of water viscosity measured for different probe sizes and different oscillation amplitudes. Measurement techniques and instrumentation were identical to those shown in Fig 3 and described in Methods. As shown the measured viscosity of water is largely independent of probe size and oscillation amplitude as expected for a Newtonian fluid. Thus, the marked dependence on these parameters in mucus reflects the lengthscale-dependent rheology and structure of the mucus rather than any systematic instrumentation or measurement errors. Error bars also show that our instrument/measurement error is ~2.6% which is well below the measured ~20% range among different data trials in mucus.
(PDF)

S2 Fig Distributions of trapped probe positions for varying probe sizes in water (left) and mucus (right). Each distribution is the cumulative laser deflection data for 10 different trapped stationary probes (color scheme as in S1 Fig), recorded at 2 kHz with a PSD for ~20 s for each probe. Each distribution is fit to a Gaussian curve with a variance *σ*^*2*^ that, as described in Refs 56 and 57, is inversely proportional to the trap stiffness *k* via the equipartition theorem (i.e. *½kσ*^*2*^ = *½k*_*B*_*T*). Because the index of refraction of the fluid can alter the trap stiffness, this calibration method, which can be carried out in the fluid of interest (i.e. mucus) is used to determine how the trap stiffness in mucus compares to that in water. The variances displayed in each plot are in units of 10^−3^ mV^2^. As shown the variance of the PSD voltage for all bead sizes in water (left) and in mucus (right) are all quite similar, within a factor of 2 of each other. The symmetry and goodness of Gaussian fits for nearly all of the distributions demonstrate that our optical trap is trapping properly and optimized for quantitative force measurements.
(PDF)

S3 Fig Diffusion coefficients of coated microspheres in mucus as compared to water. 2 μm (green) and 6 μm (blue) probes embedded in mucus and in water were imaged and tracked over time to determine the mean-squared displacements in the *x* and *y* directions. The resulting diffusion coefficients, *D*_*mucus*_ and *D*_*water*_, calculated via the Einstein relation *<Δx*^*2*^*> = <Δy>*^*2*^ = *2Dt*, were determined by tracking ~100 different probes for ~20 sec each at a frame rate of 10 fps. Error bars were determined by bootstrapping over 1000 subensembles. Measurements were carried out for probes coated with BSA, 2 kDa PEG-diamine and streptavidin (ST). Coating of carboxylated microspheres with PEG-diamine and BSA was done following the protocols published in Refs 39 and 61 which were modified from those provided by the microsphere manufacturer (Polysciences). ST-coated probes were purchased from Polysciences. Probes coated with PEG, following the cited protocols, have been shown to be mucoinert (no binding interactions with mucus) (Refs 39, 61) whereas ST is a relatively “sticky” protein that nonspecifically interacts with a range of molecules. BSA-coated probes, assumed to be mucoinert, were used in all active microrheology data shown in manuscript. As shown, the diffusion of PEG-coated and BSA-coated probes in mucus are nearly identical, suggesting similar passivation efficacy, whereas ST-coated probes show slower transport, likely due to binding interactions with the mucus. Further, the results for the BSA- and PEG-coated probes are in agreement with the measured viscosities shown in Fig 3. Namely, for 2 μm probes *D*_*mucus*_*/D*_*water*_ is ~0.59 which is consistent with the viscosity of the mucus as actively measured with 2 μm probes (~2*η*_*w*_), whereas for 6 μm probes *D*_*mucus*_*/D*_*water*_ is ~0.23 as expected provided an average measured viscosity of ~5*η*_*w*_.
(PDF)
