# Correction: Active microrheology determines scale-dependent material properties of *Chaetopterus* mucus

**DOI:** 10.1371/journal.pone.0203102

**Published:** 2018-08-23

**Authors:** W. J. Weigand, A. Messmore, J. Tu, A. Morales-Sanz, D. L. Blair, D. D. Deheyn, J. S. Urbach, R. M. Robertson-Anderson

The following information is missing from the Funding section: This study was supported by the National Science Foundation grant 1460645 (RMRA and AM).
