# Supplementary material for: Active microrheology determines scale-dependent material properties of Chaetopterus mucus
Source: PLoS One. 2017 May 31;12(5):e0176732. doi: 10.1371/journal.pone.0176732 (PMC5451080; doi:10.1371/journal.pone.0176732)
Supplement: S1 Fig — Measurement techniques and instrumentation were identical to those shown in Fig 3 and described in Methods. As shown the measured viscosity of water is largely independent of probe size and oscillation amplitude as expected for a Newtonian fluid. Thus, the marked dependence on these parameters in mucus reflects the lengthscale-dependent rheology and structure of the mucus rather than any systematic instrumentation or measurement errors. Error bars also show that our instrument/measurement error is ~2.6% which is well below the measured ~20% range among different data trials in mucus. (PDF) [file pone.0176732.s001.pdf]

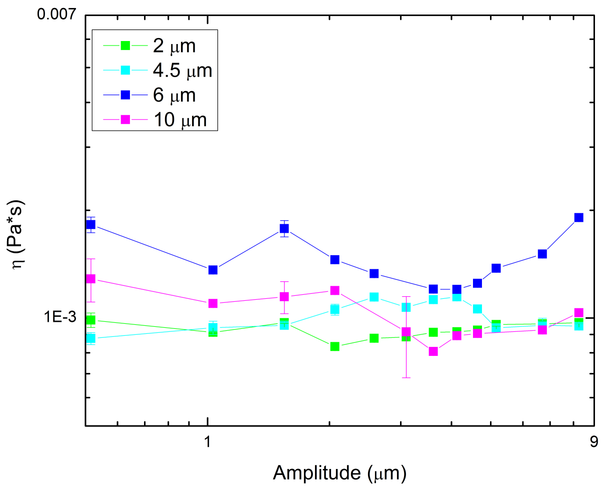

S1 Figure: Active microrheology measurements of water viscosity measured for different probe sizes and different oscillation amplitudes. Measurement techniques and instrumentation were identical to those shown in Figure 3 and described in Methods. As shown the measured viscosity of water is largely independent of probe size and oscillation amplitude as expected for a Newtonian fluid. Thus, the marked dependence on these parameters in mucus reflects the lengthscale-dependent rheology and structure of the mucus rather than any systematic instrumentation or measurement errors. Error bars also show that our instrument/measurement error is ~2.6% which is well below the measured ~20% range among different data trials in mucus.
