# Supplementary material for: Active microrheology determines scale-dependent material properties of Chaetopterus mucus
Source: PLoS One. 2017 May 31;12(5):e0176732. doi: 10.1371/journal.pone.0176732 (PMC5451080; doi:10.1371/journal.pone.0176732)
Supplement: S2 Fig — Each distribution is the cumulative laser deflection data for 10 different trapped stationary probes (color scheme as in S1 Fig), recorded at 2 kHz with a PSD for ~20 s for each probe. Each distribution is fit to a Gaussian curve with a variance σ2 that, as described in Refs 56 and 57, is inversely proportional to the trap stiffness k via the equipartition theorem (i.e. ½kσ2 = ½kBT). Because the index of refraction of the fluid can alter the trap stiffness, this calibration method, which can be carried out in the fluid of interest (i.e. mucus) is used to determine how the trap stiffness in mucus compares to that in water. The variances displayed in each plot are in units of 10−3 mV2. As shown the variance of the PSD voltage for all bead sizes in water (left) and in mucus (right) are all quite similar, within a factor of 2 of each other. The symmetry and goodness of Gaussian fits for nearly all of the distributions demonstrate that our optical trap is trapping properly and optimized for quantitative force measurements. (PDF) [file pone.0176732.s002.pdf]

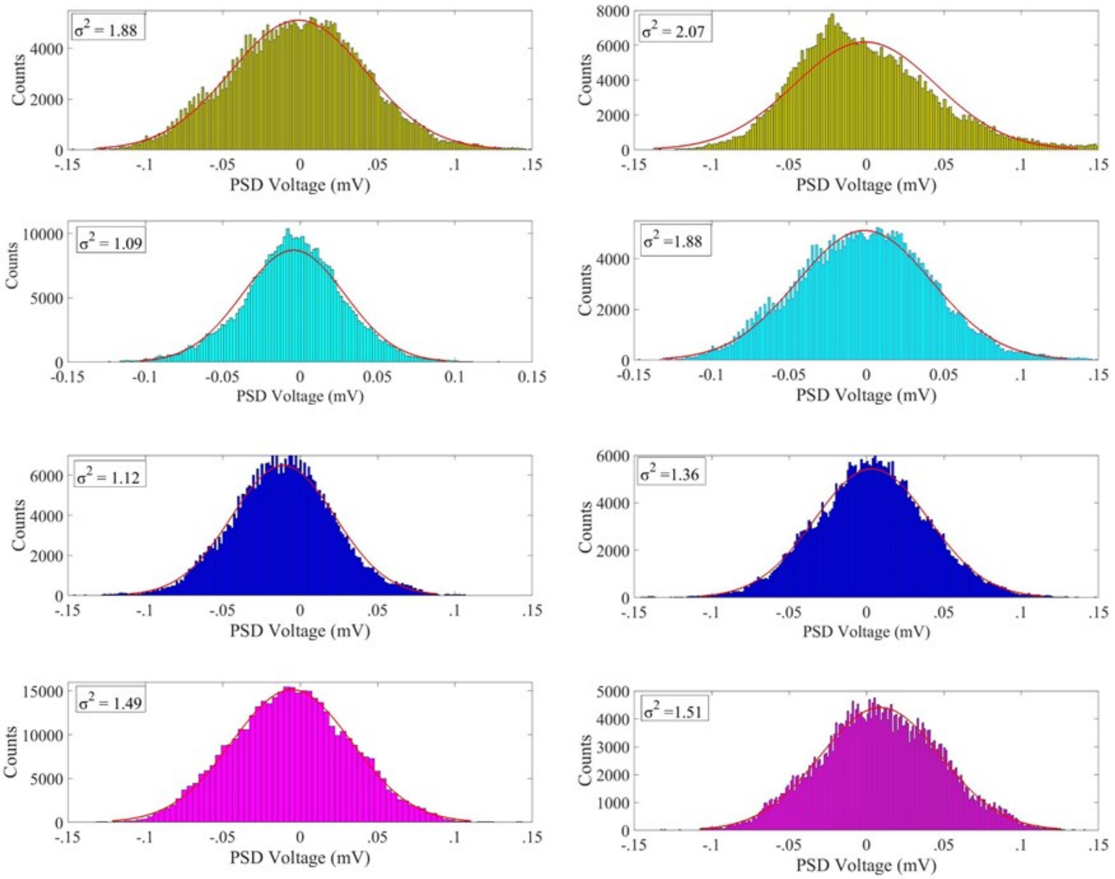

S2 Figure: Distributions of trapped probe positions for varying probe sizes in water (left) and mucus (right). Each distribution is the cumulative laser deflection data for 10 different trapped stationary probes (color scheme as in Fig SI.1), recorded at 2 kHz with a PSD for ~20 s for each probe. Each distribution is fit to a Gaussian curve with a variance σ^2 that, as described in Refs 56 and 57, is inversely proportional to the trap stiffness k via the equipartition theorem (i.e. $½k\sigma^2 = ½k_BT$). Because the index of refraction of the fluid can alter the trap stiffness, this calibration method, which can be carried out in the fluid of interest (i.e. mucus) is used to determine how the trap stiffness in mucus compares to that in water. The variances displayed in each plot are in units of 10^{-3} mV^2. As shown the variance of the PSD voltage for all bead sizes in water (left) and in mucus (right) are all quite similar, within a factor of 2 of each other. The symmetry and goodness of Gaussian fits for nearly all of the distributions demonstrate that our optical trap is trapping properly and optimized for quantitative force measurements.
