# Supplementary material for: Active microrheology determines scale-dependent material properties of Chaetopterus mucus
Source: PLoS One. 2017 May 31;12(5):e0176732. doi: 10.1371/journal.pone.0176732 (PMC5451080; doi:10.1371/journal.pone.0176732)
Supplement: S3 Fig — 2 μm (green) and 6 μm (blue) probes embedded in mucus and in water were imaged and tracked over time to determine the mean-squared displacements in the x and y directions. The resulting diffusion coefficients, Dmucus and Dwater, calculated via the Einstein relation <Δx2> = <Δy>2 = 2Dt, were determined by tracking ~100 different probes for ~20 sec each at a frame rate of 10 fps. Error bars were determined by bootstrapping over 1000 subensembles. Measurements were carried out for probes coated with BSA, 2 kDa PEG-diamine and streptavidin (ST). Coating of carboxylated microspheres with PEG-diamine and BSA was done following the protocols published in Refs 39 and 61 which were modified from those provided by the microsphere manufacturer (Polysciences). ST-coated probes were purchased from Polysciences. Probes coated with PEG, following the cited protocols, have been shown to be mucoinert (no binding interactions with mucus) (Refs 39, 61) whereas ST is a relatively “sticky” protein that nonspecifically interacts with a range of molecules. BSA-coated probes, assumed to be mucoinert, were used in all active microrheology data shown in manuscript. As shown, the diffusion of PEG-coated and BSA-coated probes in mucus are nearly identical, suggesting similar passivation efficacy, whereas ST-coated probes show slower transport, likely due to binding interactions with the mucus. Further, the results for the BSA- and PEG-coated probes are in agreement with the measured viscosities shown in Fig 3. Namely, for 2 μm probes Dmucus/Dwater is ~0.59 which is consistent with the viscosity of the mucus as actively measured with 2 μm probes (~2ηw), whereas for 6 μm probes Dmucus/Dwater is ~0.23 as expected provided an average measured viscosity of ~5ηw. (PDF) [file pone.0176732.s003.pdf]

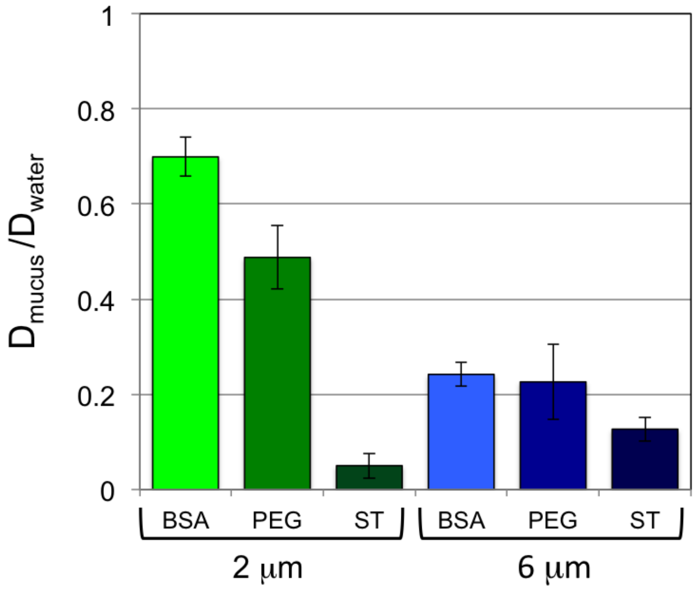

S3 Figure: Diffusion coefficients of coated microspheres in mucus as compared to water. 2 μm (green) and 6 μm (blue) probes embedded in mucus and in water were imaged and tracked over time to determine the mean-squared displacements in the *x* and *y* directions. The resulting diffusion coefficients, D_{mucus} and D_{water}, calculated via the Einstein relation $<\Delta x^2>=<\Delta y>^2=2Dt$, were determined by tracking ~100 different probes for ~20 sec each at a frame rate of 10 fps. Error bars were determined by bootstrapping over 1000 subensembles. Measurements were carried out for probes coated with BSA, 2 kDa PEG-diamine and streptavidin (ST). Coating of carboxylated microspheres with PEG-diamine and BSA was done following the protocols published in Refs 39 and 61 which were modified from those provided by the microsphere manufacturer (Polysciences). ST-coated probes were purchased from Polysciences. Probes coated with PEG, following the cited protocols, have been shown to be mucoinert (no binding interactions with mucus) (Refs 39, 61) whereas ST is a relatively "sticky" protein that nonspecifically interacts with a range of molecules. BSA-coated probes, assumed to be mucoinert, were used in all active microrheology data shown in manuscript. As shown, the diffusion of PEG-coated and BSA-coated probes in mucus are nearly identical, suggesting similar passivation efficacy, whereas ST-coated probes show slower transport, likely due to binding interactions with the mucus. Further, the results for the BSA- and PEG-coated probes are in agreement with the measured viscosities shown in Figure 3. Namely, for 2 μm probes D_{mucus}/D_{water} is ~0.59 which is consistent with the viscosity of the mucus as actively measured with 2 μm probes (~$2\eta_w$), whereas for 6 μm probes D_{mucus}/D_{water} is ~0.23 as expected provided an average measured viscosity of ~$5\eta_w$.
